# Plasma Spectrum Diagnosis and Cleaning Quality Analysis of Laser Cleaning of Marine Biofilms on Aluminum Alloy Surfaces

**DOI:** 10.3390/ma18081843

**Published:** 2025-04-17

**Authors:** Zhenglong Lei, Qiang Meng, Xinrui Zhang, Xudong Li, Chen Wang, Bao Zhao

**Affiliations:** 1 State Key Laboratory of Precision Welding and Joining of Materials and Structures, Harbin Institute of Technology, Harbin 150080, China; m17835852521@163.com (Q.M.); 20b909125@stu.hit.edu.cn (X.Z.); 23b909179@stu.hit.edu.cn (X.L.); wangchen22527@163.com (C.W.); 2 Suzhou Research Institute, Harbin Institute of Technology, Suzhou 215104, China; 3 China Machinery Group Harbin Welding Research Institute Co., Ltd., Harbin 150080, China; zhaobaojx@sina.com

**Keywords:** laser cleaning, marine biofilm layer, LIBS, aluminum alloy, cleaning quality, plasma spectra

## Abstract

Surface quality monitoring has become increasingly important in the laser cleaning process. Currently, most research focuses on cleaning contaminants such as oxides and paints, while studies on the cleaning of marine biofilm layers from metal surfaces remain limited. This paper presents real-time monitoring of nanosecond pulsed laser cleaning of marine biofilm layers on aluminum alloy surfaces using laser-induced breakdown spectroscopy (LIBS). The plasma spectra of different microbial layers during cleaning were collected to analyze the variations in characteristic elements. Regression fitting techniques were used to analyze the evolution of plasma spectra in the long-wave band and at specific wavelengths, establishing a relationship between spectral signals and cleaning effectiveness. After cleaning, energy dispersive spectroscopy (EDS) characterization was performed on the sample surface to verify the changes in elemental composition during the cleaning process of different marine biofilm layers. Additionally, the plasma spectra corresponding to the optimal cleaning process for each microbial layer were defined as the “reference spectrum”. The Pearson correlation coefficient between random spectra and the “reference spectrum” was calculated to determine the optimal cleaning process. The highest correlation results were found to predict the optimal cleaning parameters with a relative error between 0.9% and 3.8% when compared to experimentally measured values. The feasibility of LIBS technology for monitoring the laser cleaning process of marine biofilm layers on metal surfaces was validated in this study, and a theoretical foundation was provided for the future use of LIBS in enabling intelligent feedback control of the laser cleaning process.

## 1. Introduction

In marine engineering, metal equipment is susceptible to contamination by marine microorganisms during prolonged use, leading to the formation of biofilms. These biofilms not only affect the functionality and operational efficiency of the equipment but also induce corrosion, thereby accelerating the oxidation and deterioration of the metal. According to statistics, the direct economic losses caused by marine biofouling on global marine equipment reach billions of dollars. In the United States, the annual financial loss due to marine microorganism fouling amounts to USD 3 to 5 billion, while in China, the yearly economic loss caused by marine microorganism fouling is approximately RMB 50 billion. Indirect energy losses are also significant; for example, the related costs of the DDG-51 fleet amount to about USD 56 million annually [1,2,3]. Traditional methods for removing biofilm layers include mechanical, chemical, and physical cleaning. However, these methods are inefficient, cause environmental pollution, and are difficult to apply to precision equipment. In contrast, laser cleaning offers advantages such as minimal material damage, high efficiency, and precision, enabling the removal of unevenly growing contaminants, such as marine microorganisms, while preserving the integrity of the metal surface. Therefore, laser cleaning is an effective method for removing biofilm layers from metal surfaces.

Appropriate inspection methods must be employed to ensure the quality of laser cleaning, such as visual inspection, roughness measurement, and optical inspection, but they suffer from low precision, poor timeliness, and subjective results. Furthermore, these methods have limited capability to detect contaminant residues during the cleaning process, making it challenging to analyze multiple contaminants. They also exhibit low sensitivity and are influenced by human factors. Laser-induced breakdown spectroscopy (LIBS), an advanced spectral analysis technique, enables real-time monitoring of the quality of laser cleaning. LIBS not only accurately identifies the types of contaminants but also performs both qualitative and quantitative analyses. More importantly, it assesses the extent of damage to the substrate surface, offering a comprehensive evaluation of the laser cleaning process. Compared to traditional inspection methods, LIBS offers significant advantages in terms of being real-time, non-destructive, and requiring no direct intervention on the substrate. These advantages make LIBS particularly suitable for inspecting and monitoring the cleaning effect on material surfaces during precision cleaning processes. This technology does have certain limitations in its use, such as strong environmental interference and insufficient signal resolution. However, these issues can be addressed in experiments through methods such as background subtraction and baseline correction.

A. A. Voznesenskaya from Vladimir State University [4] conducted spectroscopic monitoring of laser cleaning on aluminum alloy surfaces coated with primer paint. The study determined the wavelength range characterizing the removal of the paint layer, identified atomic and ionic lines in the spectra, and used a set of Al and Cr spectral lines as criteria for assessing complete primer removal and ensuring that the laser interacted only with the surface layer. Xing Li [5] from Beihang University investigated the laser cleaning of the oxide layer on stainless steel surfaces. The study found that as laser power increased, spectral signals exhibited characteristic peaks corresponding to the substrate and the oxide layer. X-ray diffraction (XRD) and post-cleaning three-dimensional surface morphology analysis were combined to examine elemental changes during the cleaning process. The study also utilized the relative intensity ratios (RIRs) of two characteristic peaks, Fe I-520.9 nm and Cr I-589.2 nm, to qualitatively evaluate the cleaning process. Kangxi Chen [6] from Sichuan University combined LIBS with X-ray spectroscopy to calculate plasma density and temperature using the LIBS spectra of elemental O and Ti. The study found that more chemical bonds in the paint were broken as plasma electron density increased. Additionally, changes in the relative ratios of elemental Ti to C and Al in the EDS analysis indicated an improvement in paint removal efficiency.

Currently, it has been found that the monitoring of spectral signals for laser cleaning mainly relies on laser-induced breakdown spectroscopy (LIBS) [4,5,6,7,8,9,10,11,12,13,14,15,16,17,18,19]. However, most studies focus on uniformly distributed contaminants, such as paint and oxides, making cleaning quality analysis relatively straightforward. In contrast, research on laser cleaning quality inspection for marine biofilm layers on metal surfaces remains limited. This paper captures plasma spectral signals during the laser cleaning of marine biofilm layers on aluminum alloy surfaces. It analyzes the evolution of different spectral lines, examines the variation in characteristic elements corresponding to various cleaning substances throughout the laser process, and assesses the cleaning effect using characterization methods. Additionally, the feasibility of spectral signal detection is verified, and the cleaning effect is further evaluated using the “reference spectrum”.

## 2. Materials and Methods

The experimental base material is 6061 aluminum alloy, with 150 mm × 40 mm × 2 mm dimensions. Table 1 shows its main chemical composition.

**Figure 1:**
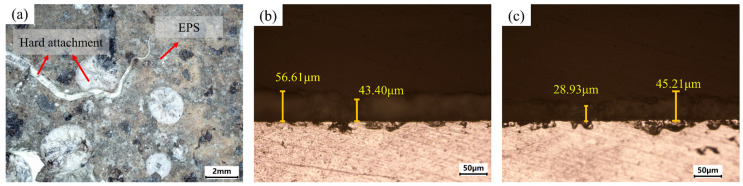
Aluminum alloy surface microbial macro-morphology and cross-section thickness: (**a**) macro-morphology, (**b**) hard attachments cross-section thickness, (**c**) and EPS layer cross-section thickness.

Before the experiment, the aluminum alloy specimen was wiped with alcohol, punched, strung together with wire, and suspended approximately 2 m underwater beneath a ship in the Yellow Sea. After three months of immersion, the specimen was removed. The surface morphology, shown in Figure 1a, revealed the growth of many white marine microorganisms. These microorganisms were distributed unevenly and classified as hard attachments [20]. The underlying gray-black layer primarily consisted of extracellular polymeric substances (EPSs) [20], which are mainly composed of microbial secretions. This layer densely covered the entire plate surface and exhibited a more uniform distribution than the hard attachments.

The cross-sectional thicknesses of typical hard attachments and EPS layers were examined, as shown in Figure 1b,c. The results indicated that the average thickness of hard attachments exceeded that of the EPS layers. Based on multi-point statistical analysis, the thickness of hard attachments ranged from 40 to 65 μm, while the EPS layers varied between 20 and 50 μm. To control the experimental variables and ensure the consistency of the cleaning difficulty of the same type of microorganisms, regions with hard attachment thicknesses of approximately 50 μm and EPS layer thicknesses of about 35 μm were selected for cleaning in the experiments.

**Figure 2:**
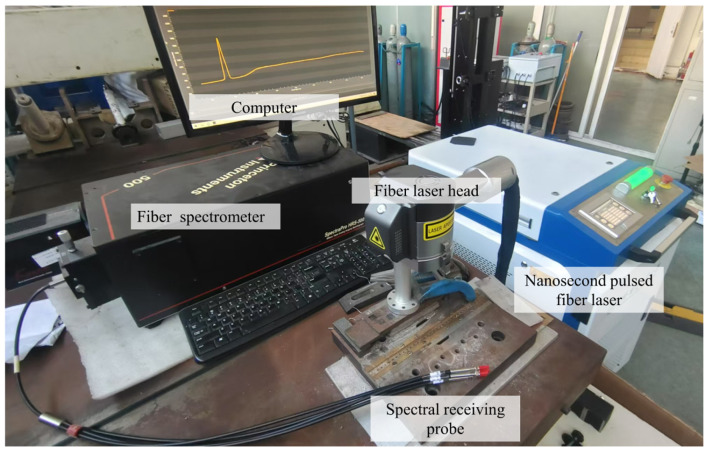
Laser and spectral acquisition system.

The laser cleaning equipment utilized an IPG 200 W nanosecond pulsed fiber laser with a maximum output power of 200 W and a laser wavelength of 1064 nm. The pulse width ranged from 30 to 240 ns. The laser beam passed through the galvanometer scanning head, following an ‘S’ trajectory; the rectangular scanning path of 15 mm × 15 mm was used for laser cleaning. The SpectraPro HRS-500 fiber optic spectrometer, manufactured by Princeton Instruments (USA), was employed to monitor the plasma during the cleaning process. The wavelength range of the spectrometer is from 300 nm to 800 nm, with a measurement accuracy of 0.01 nm. When a laser interacts with a surface, it generates plasma containing elements from contamination layers and the substrate. By analyzing the emission spectra, specific characteristic spectral lines of contaminants (e.g., oxides, marine biofilms) can be detected. The presence (or disappearance) of specific elements in the plasma emission indicates whether the contaminants have been successfully removed. The cleaning equipment and spectral acquisition system are shown in Figure 2, with the fiber optic probe positioned 80 mm away from the workpiece in the horizontal direction.

The JSZ7 digital microscope, manufactured by HST in Vietnam, was used to observe the macroscopic morphology of the cleaned metal surface in order to support the analysis of the relationship between the plasma spectra and the cleaning effect. The Quanta 200F scanning electron microscope, manufactured by FEI Company (USA), was used to observe the micro-morphological characteristics of the surfaces before and after cleaning. During SEM analysis, prolonged exposure to a high-energy electron beam may degrade the organic biofilm due to the poor conductivity of the marine biofilm. Therefore, gold sputter coating was applied to enhance surface conductivity while preserving the structural integrity of the biofilm. Additionally, the scanning electron microscope with an energy dispersive spectroscopy (EDS) analyzer was used to examine the types, contents, and distributions of micro-area elements on the substrate surface before and after laser cleaning.

## 3. Results and Discussion

### 3.1. Compositional Analysis and Elemental Identification of Biofilm Layers on Aluminum Alloy Surfaces

Based on the analysis in Section 2, the aluminum alloy surface was covered by two distinct biofilm layers with different compositions after immersion. Real-time monitoring of the cleaning process using LIBS technology requires the extraction of primary elemental composition signals from the biofilm layers and the identification of characteristic elements that differentiate microbial layers.

**Figure 3:**
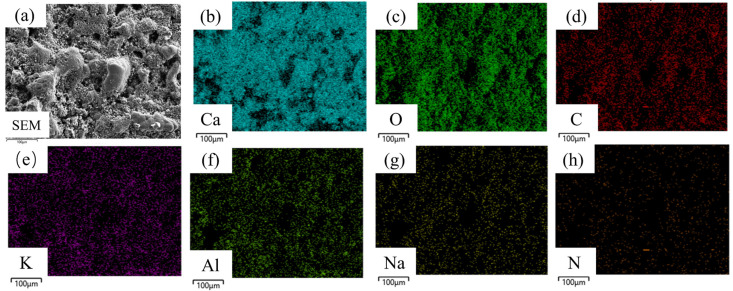
Elemental distribution of the surface hard attachments. (**a**) SEM; (**b**) Ca; (**c**) O; (**d**) C; (**e**) K; (**f**) Al; (**g**) Na; (**h**) N.

**Figure 4:**
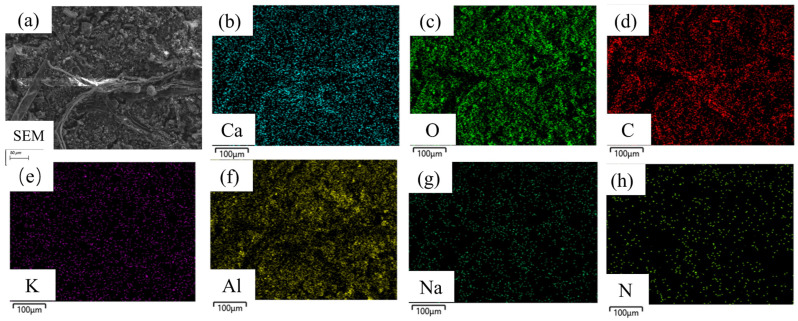
Elemental distribution of the underlying extracellular polymeric substances (EPS layers). (**a**) SEM; (**b**) Ca; (**c**) O; (**d**) C; (**e**) K; (**f**) Al; (**g**) Na; (**h**) N.

Several representative locations on the hard attachment layers and EPS layers were selected for energy-dispersive spectroscopy (EDS) surface scanning to facilitate the achievement of the analysis objective. The analysis focused on the elements C, O, Al, K, Ca, Na, and N. The elemental distribution at these selected locations is shown in Figure 3 and Figure 4, where differences in light and dark shading represent variations in element content.

As shown in Figure 3, the distribution areas of various elements in the surface hard attachments largely overlapped, with high concentrations of Ca (13.03 At.%), O (59.79 At.%), and C (24.94 At.%). In contrast, only a few localized areas exhibited significantly lower concentrations of these elements. The significant presence of calcium (Ca) and carbon (C) in the EDS spectra indicated the existence of calcified biological materials, such as algae with mineralized cell walls. In addition to Ca and C, the detection of oxygen (O) further confirmed the presence of calcium carbonate (CaCO_3_). These results suggest that the hard deposits contained a substantial amount of micro-calcified algae, as CaCO_3_ is a major component of calcareous algae, which converted CO_2_ into CO_3_^2−^, reacting with Ca^2^^+^ to form CaCO_3_ [21]. The trace inorganic salt elements K (0.66 At.%) and Na (0.57 At.%) were relatively evenly distributed. Additionally, a small amount of the matrix element Al (0.91 At.%) was detected and showed a uniform distribution. These findings indicate that the hard attachments mainly consisted of micro-calcified algae, with minor amounts of inorganic salts and aluminum oxidation products.

As shown in Figure 4, the EPS layers uniformly covered the substrate surface. The organic components C (48.26 At.%) and O (41.49 At.%) were the most abundant, accounting for nearly 90% of the total atomic composition. The remaining inorganic salt components, including Ca (0.2 At.%), K (1.03 At.%), and Na (0.38 At.%), were sparsely distributed. The distribution of Al (5.61 At.%) overlapped with that of C and O, and its content increased compared to the hard attachments. The higher aluminum content in the EPS layers compared to the hard attachments can be attributed to its distribution and structural characteristics. The EPS layers were located on the substrate surface and had a relatively small thickness. In contrast, the hard attachment layers were predominantly found on the outermost surface, with some regions adhering to the EPS layers. As a result, aluminum from the substrate was more readily incorporated into the densely distributed EPS layers. In contrast, the incorporation of substrate-derived aluminum into the hard attachment layers was relatively limited. The analysis indicates that the EPS layers primarily consisted of organic components C and O (nearly 90%), with minor amounts of aluminum oxides and trace inorganic salts (Ca, K, and Na) interspersed throughout [22].

**Figure 5:**
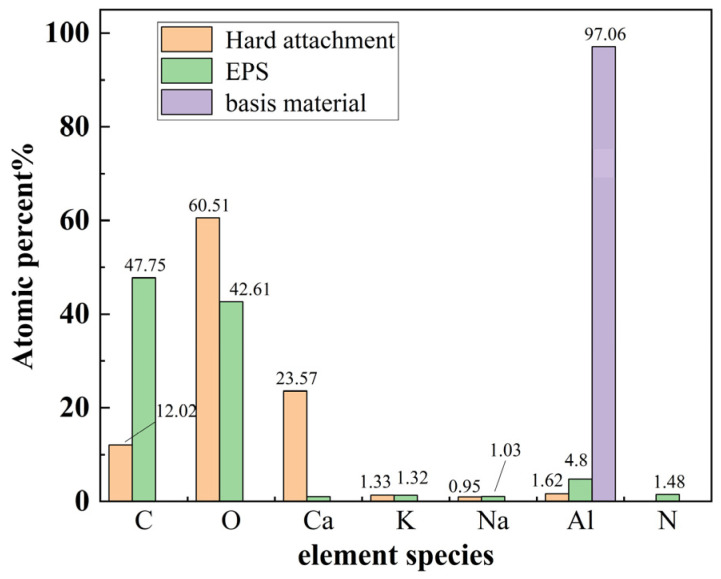
Percentage of elemental composition of different marine biofilm layers as well as substrate aluminum sheets.

To compare the differences in elemental composition between the hard attachments and the EPS layers and to identify characteristic elements (hereafter referred to as *CE*) that represent each layer, EDS scans were conducted on multiple samples of the hard attachments, the EPS layers, and the aluminum alloy substrate. The scan results were averaged, and the percentage of each element was quantified, as shown in Figure 5. The figure illustrates that the hard attachments contained the highest concentration of O, followed by Ca; the EPS layers consisted almost entirely of C and O, with minimal amounts of other elements; and the aluminum alloy substrate had the highest concentration of Al. Therefore, Ca was selected as the *CE* for the hard attachments, Al as the *CE* for the substrate, and C and O as the *CE* for the EPS layers. Although C and O were also present in high amounts in the hard attachments, the extremely high concentrations of these two elements in the EPS layers, combined with the very low concentrations of other elements (e.g., Ca and Al), allowed clear differentiation of the EPS layers from the hard attachments.

### 3.2. Analysis of Peak Intensities and Spectral Characteristics of Spectral Features in Different Marine Biofilm Layers

**Figure 6:**
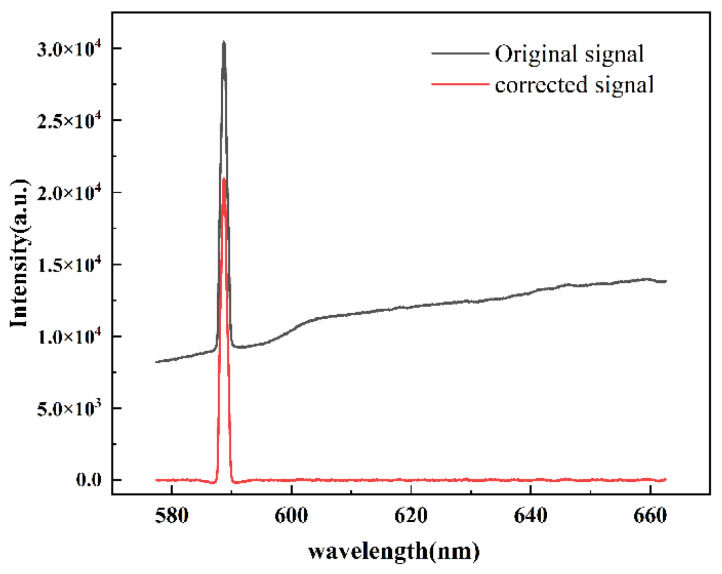
Baseline correction of raw signal by airPLS.

To eliminate background spectral interference from the external environment, the Adaptive Iterative Re-weighted Penalized Least Squares method (airPLS) was applied for baseline correction. This method effectively removes background interference and improves signal accuracy before acquiring the spectral data in each experiment. As shown in Figure 6, the airPLS method achieved baseline correction without affecting the actual spectral features.

As discussed in Section 3.1, the hard attachments and EPS layers differed in distribution and elemental composition. To compare the differences in the types and intensities of the characteristic peaks of the spectral signals when cleaning different biofilm layers, aluminum alloy surfaces were cleaned under different energy densities for hard attachments and EPS layers to obtain corresponding spectral signals. Calibration was then performed using the NIST Atomic Spectroscopy Database.

**Figure 7:**
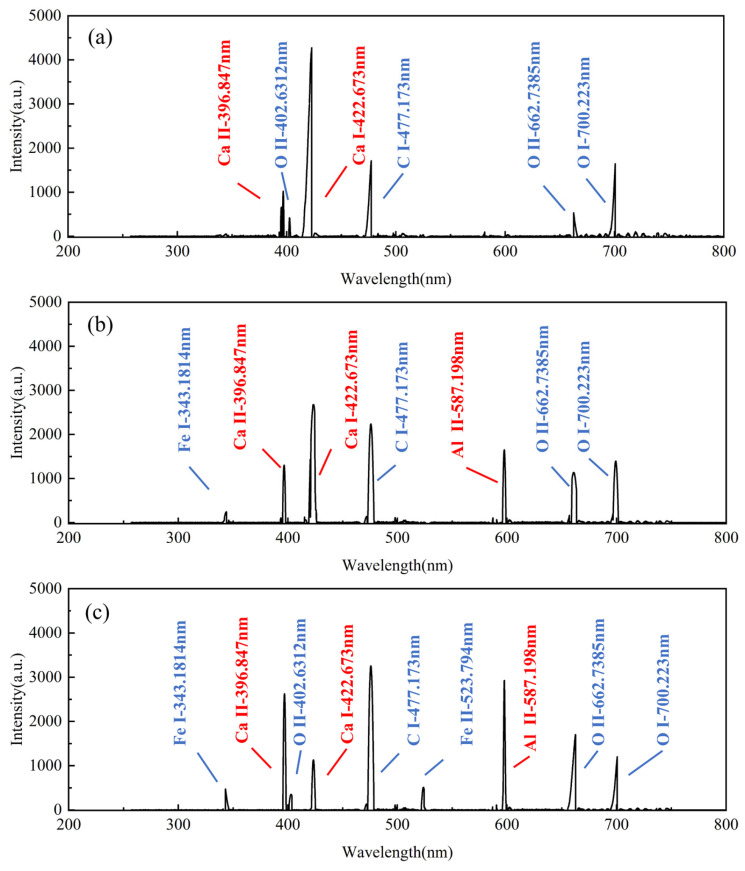
Calibration results of plasma spectra for cleaning hard attachments at different energy densities: (**a**) 5.5 J/cm^2^, (**b**) 9.4 J/cm^2^, and (**c**) 15.5 J/cm^2^ (Ca I-422.673 nm, Ca II-396.847 nm, and Al II-587.198 nm are key spectral lines of interest).

**Figure 8:**
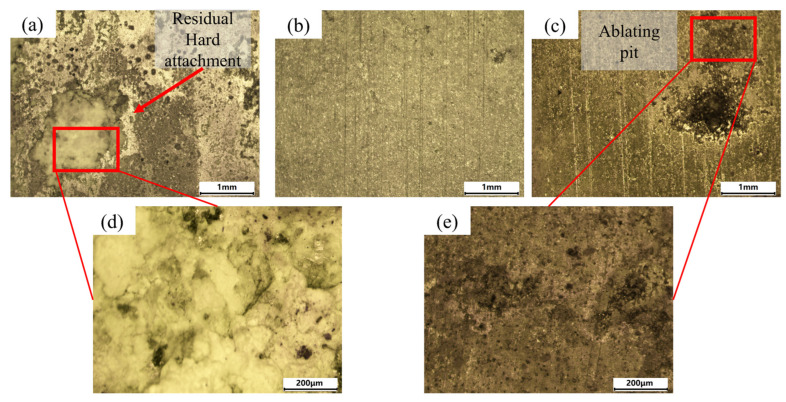
Macroscopic morphology of the surface after cleaning the hard attachments: (**a**) 5.5 J/cm^2^, (**b**) 9.4 J/cm^2^, and (**c**) 15.5 J/cm^2^. (**d**) Magnified view of the residual hard attachment. (**e**) Magnified view of the oxidized region.

The spectral signal calibration results for cleaning the hard attachments at different energy densities are shown in Figure 7. The macroscopic morphology of the cleaned surface is presented in Figure 8. At a low energy density (5.5 J/cm^2^), the calibration results exhibited the characteristic elemental peaks Ca I-422.673 nm and Ca II-396.847 nm for the hard attachments. The intensity of the atomic spectral line (Ca I) was higher than that of the ionization spectral line (Ca II), which exhibited lower intensity. The weaker plasma inverse bremsstrahlung absorption effect can be attributed to the lower energy density of the laser, which also causes the spectrometer to detect ion spectral signals weakly, with lower intensity [22]. In addition, atomic spectral lines such as O I-700.223 nm and C I-477.173 nm, as well as O II-662.7385 nm and O II-402.6312 nm, were observed. As discussed in Section 3.1, although the contents of O and C elements in the hard attachments were higher than that of Ca, the intensities of their spectral lines remained lower than that of the characteristic Ca I line. As the atomic number increases, the atomic radius also increases, which leads to reduced attraction between the nucleus and the outermost valence electrons. Consequently, the first excitation and ionization potentials are lower, making excitation and ionization easier and resulting in weaker spectral line intensities [23]. The shift from atomic to ionic spectral lines reflects increasing laser–material interaction intensity. At low energy densities, atomic lines dominate, indicating mild excitation mainly affecting surface contaminants. As energy density rises, ionic lines emerge, signifying stronger ablation and plasma formation that enhance cleaning. However, if this transition involves substrate elements, it may indicate potential substrate damage and reduced cleaning efficiency. As shown in Figure 8a,d, large pieces of hard attachment residue remained on the surface. At this energy density, although the laser can stimulate the atomic and ion spectral lines associated with hard attachments, the intensity is low and the cleaning effect is poor; at this point, the atomic percentage of Al was 17.59%.

As the energy density increased (9.4 J/cm^2^), new spectral lines such as Fe I-343.1814 nm and Al II-587.198 nm appeared, in addition to the previous spectral lines, indicating that the laser had interacted with the surface of the substrate. The intensity of Ca I-422.673 nm decreased, while the intensity of Ca II-396.847 nm increased. The higher laser energy density enhanced plasma ionization, resulting in a gradual transition of spectral lines from atomic to ionic [24]. Figure 8b shows that the hard surface attachments were almost completely removed, exposing the substrate, which exhibited isotropic linear streak traces under the action of the laser. The appearance of a new spectral line (Al II) and the increased intensity of the original spectral lines indicated that a higher energy density removed more hard attachments, demonstrating an improvement in the cleaning effect; at this point, the atomic percentage of Al was 44.32%.

The energy density was further increased (15.5 J/cm^2^), and the intensity of the existing spectral lines continued to increase. A greater variety of spectral lines and higher intensities indicated the removal of a larger amount of hard surface attachments. As shown in Figure 8c,e, the entire surface underwent laser oxidative ablation, with some areas even exhibiting ablation pits. At high energy densities, the heat generated by the laser exceeds the material’s ability to dissipate it, resulting in excessive heating and thermal damage, which leads to localized overheating and ablation. High laser energy generates dense laser-induced plasma (LIP) above the surface. The high-energy plasma exerts pressure on the substrate, causing additional shockwave-induced material ejection, which leads to deeper pit formation. The substrate material, having been submerged in seawater for an extended period, undergoes pitting corrosion. Corroded aluminum alloys typically exhibit localized changes, such as pores and microcracks, which reduce thermal conductivity. The lowered thermal conductivity allows the laser energy to concentrate more easily on the surface, exacerbating ablation and resulting in the formation of ablation pits. It is evident that excessive laser energy caused damage to the substrate, leading to a poor cleaning effect; at this point, the atomic percentage of Al was 46.33%.

**Figure 9:**
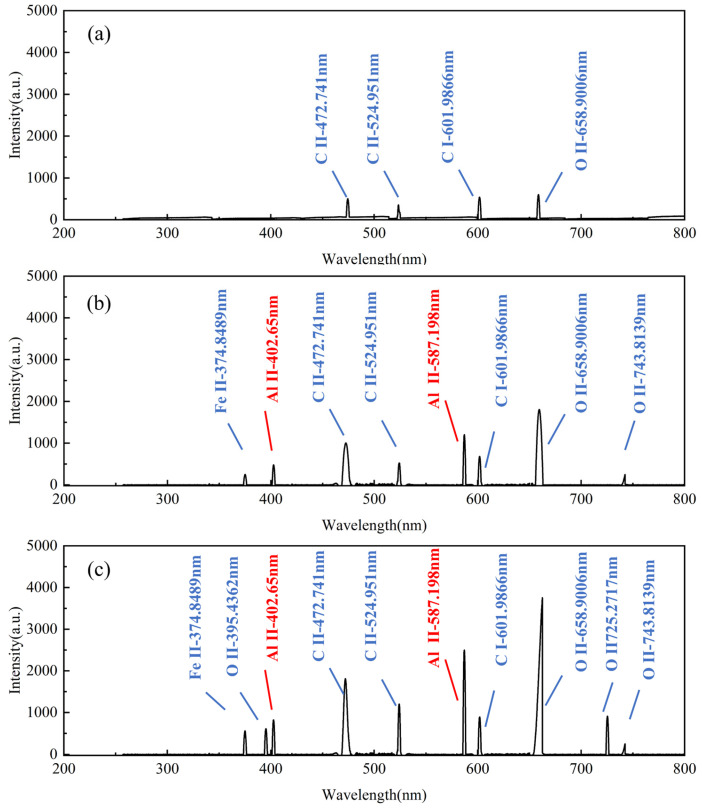
Calibration results of cleaning EPS plasma spectra at different energy densities: (**a**) 5.53 J/cm^2^, (**b**) 7.7 J/cm^2^, and (**c**) 11.4 J/cm^2^ (C II-472.741 nm, O II-658.9006 nm, and Al II-587.198 nm are key spectral lines of interest).

**Figure 10:**
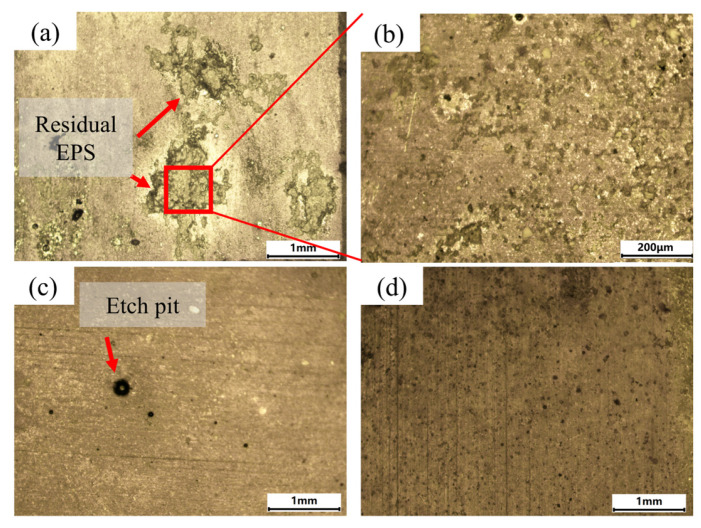
Macroscopic morphology of the surface after cleaning the EPS layers: (**a**) 5.5 J/cm^2^, (**b**) magnified view of the residual EPS layers (**c**) 7.7 J/cm^2^, and (**d**) 11.4 J/cm^2^.

The results of spectral signal calibration for cleaning the EPS layers at different energy densities are presented in Figure 9. In contrast, the macroscopic morphology of the cleaned surface is shown in Figure 10. At a low laser energy density (5.5 J/cm^2^), only a few characteristic spectral lines of C and O elements appeared, and the spectral intensity remained weak. As shown in Figure 10a,b, residual EPS layers remained in certain areas of the surface after cleaning. By analyzing the post-cleaning morphology and spectral results at low laser energy densities, only part of the EPS layer was removed, resulting in the appearance of low-intensity characteristic spectral lines of C and O in the plasma spectra, indicating a poor cleaning effect at this point, with the atomic percentage of Al at 22.29%.

When the laser energy density was increased (7.7 J/cm^2^), the peak intensity of each spectral line also increased. In addition to the previously observed spectral lines, new characteristic lines of Al and Fe elements also appeared, which suggests that the removal of the surface EPS layer significantly improved with increasing laser energy density, accompanied by an enhancement in the intensity of the characteristic spectral lines of elements C and O. Meanwhile, the laser partially interacted with the substrate surface. As shown in Figure 10c, the EPS layers were almost completely removed, exposing the metal substrate. Additionally, black seawater corrosion pits were visible. The black corrosion pits adhered to the substrate surface are the initial corrosion sites formed during immersion. Their exposure indicates the removal of the EPS layers, revealing the substrate surface, indicating an effective cleaning outcome; at this point, the atomic percentage of Al was 44.36%.

When the laser energy density was further increased (11.4 J/cm^2^), the intensity of each plasma spectral line significantly increased, particularly the characteristic spectral lines of O II-658.9006 nm and Al II-587.198 nm. This increase in intensity suggests an intensified direct interaction of the laser with the substrate. Additionally, the intensity of the previously observed characteristic spectral lines of oxygen increased markedly, and new oxygen spectral lines at different wavelengths emerged. In combination with Figure 10d, it was observed that the overall darkening of the substrate surface indicated the occurrence of an oxidation reaction under laser irradiation, leading to the formation of aluminum oxide. The oxidation phenomenon led to an increase not only in the intensity of the oxygen element’s characteristic spectral lines but also in their variety. These findings suggest that the high laser energy density induced some degree of substrate damage, ultimately leading to poor cleaning results; at this point, the atomic percentage of Al was 48.41%.

### 3.3. Analysis of the Intensity of Characteristic Spectral Lines in Specific Bands and Characterization of the Corresponding Cleaning Effect

As shown in Section 3.2, when cleaning the biofilm layers at different laser energy densities, the plasma will radiate different types and intensities of characteristic spectral lines in the 300 nm–800 nm band. The spectral lines exhibit different variation trends; some show significant amplitude changes, whereas others display only minor fluctuations. To comprehensively analyze the relationship between spectral line intensity and cleaning effectiveness, the variations in spectral line intensity with energy density across specific wavelength bands for the two types of microbial layers were quantified separately, and regression fitting equations were established. Additionally, EDS spectral scanning was conducted on the cleaned surface to analyze and verify changes in elemental composition after cleaning. In this study, a nonlinear regression using the Logistic function was employed for curve fitting. The standard form of the equation is shown in Equation (1):

(1) $$F(x)=\frac{A_{1}-A_{2}}{1+e^{\frac{\left(x-x_{0}\right)}{p}}}+A_{2}$$

**Figure 11:**
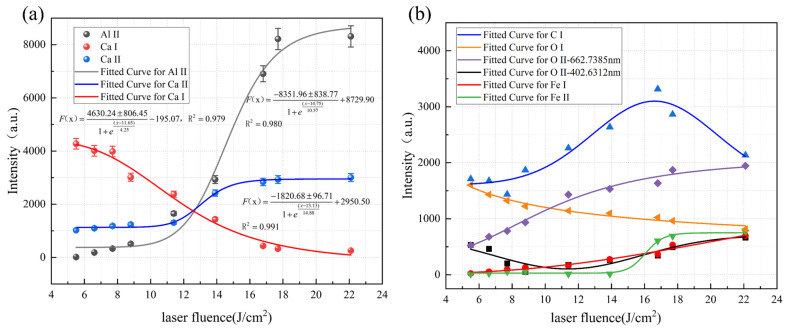
Intensity changes in characteristic spectral lines of the cleaned hard attachments. (**a**) Key spectral lines. (**b**) Non-key spectral line (Symbols of different colors and shapes represent different characteristic peaks, as indicated in the legend).

The regression equations of spectral line intensity and energy density for cleaning hard attachments are shown in Figure 11. Figure 11a illustrates that the intensities of the Ca I-422.673 nm, Ca II-396.847 nm, and Al II-587.198 nm spectral lines exhibited significant variations with changes in energy density, showing a distinct monotonic relationship. Furthermore, Ca and Al were characteristic elements representing the hard attachment layer and the substrate, respectively. However, as shown in Figure 11b, the intensity of the remaining spectral lines, such as C and O, fluctuated less with changes in energy density and was unstable. These spectral lines varied only slightly and were easily affected by the surface conditions, limiting their ability to reflect the cleaning state accurately. To more accurately assess the cleaning effectiveness through plasma spectroscopy, the spectral lines Ca I-422.673 nm, Ca II-396.847 nm, and Al II-587.198 nm (hereafter referred to as Ca I, Ca II, and Al II) were selected as key spectral lines for analysis.

As shown in Figure 11a, the intensities of the Ca II and Al II spectral lines increased with energy density. In contrast, the Ca I line exhibited higher intensity at lower energy densities, which was consistent with the results presented in Section 3.2, which indicated that when the hard attachments were not completely cleaned, the Ca I atomic spectral line was stronger. As the energy density increased, the degree of ionization rose, leading to a transition from atomic to ionic spectral lines. Additionally, as the hard attachments were removed and the laser began to act on the substrate surface, the intensity of the Al II spectral line increased sharply around 10 J/cm^2^. At approximately 18 J/cm^2^, the intensities of all three spectral lines stabilized.

**Figure 12:**
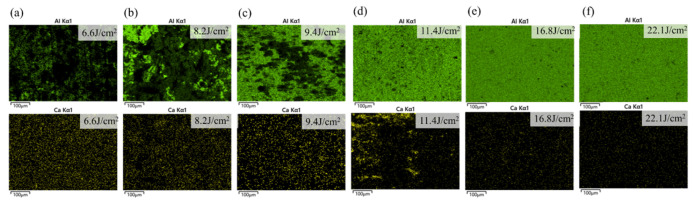
EDS scanning results of Al and Ca elements on the surface of the hard attachments after cleaning with different energy densities. (**a**) 6.6 J/cm^2^; (**b**) 8.2 J/cm^2^; (**c**) 9.4 J/cm^2^; (**d**) 11.4 J/cm^2^; (**e**) 16.8 J/cm^2^; (**f**) 22.1 J/cm^2^.

The EDS surface scans of Al and Ca after cleaning are shown in Figure 12. At low energy densities, the Al content was relatively low (13.66 At.%), with the majority of the surface being occupied by other elements. Similarly, the Ca content was also low (1.53 At.%), corresponding to the higher intensity of Ca I spectral lines observed at low energy densities in Figure 11a, which suggested that the effective removal of the superficial Ca-rich hard attachments occurred under laser irradiation at a low energy density. When the energy density reached 11.4 J/cm^2^, Al covered more than half of the surface, indicating that most of the substrate had been exposed, demonstrating a good cleaning effect; at this point, the atomic percentage of Al was 45.26%. As shown in Figure 11a, the intensity of the Al II spectral line began to increase sharply at this stage. When the energy density reached 22.11 J/cm^2^, Al was nearly uniformly distributed across the entire surface (48.27 At.%), while the Ca content decreased to an almost negligible level (0.12 At.%), indicating that the substrate material is fully exposed at this stage, and some areas have been damaged under high energy density. Additionally, the EDS scans reveal that the Al element displayed a wide range of color intensity variations, whereas the Ca element exhibited a much smaller range of changes. This observation aligned with the corresponding variations in Al and Ca spectral line intensities shown in Figure 11a.

**Figure 13:**
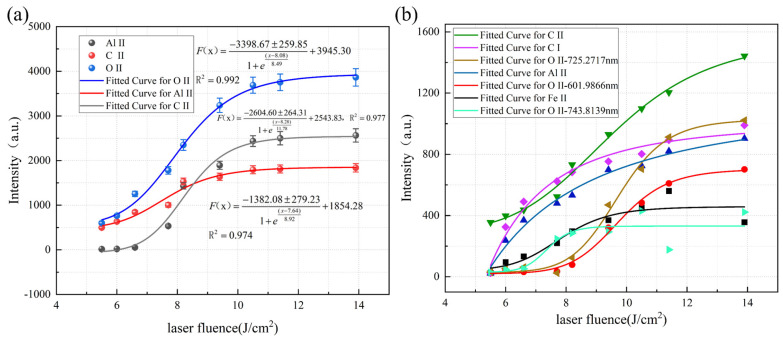
Intensity changes in characteristic spectral lines of the cleaned EPS layers. (**a**) Key spectral lines. (**b**) Non-key spectral line (Symbols of different colors and shapes represent different characteristic peaks, as indicated in the legend).

The regression equations of spectral line intensity and energy density for cleaning EPS layers are shown in Figure 13. As shown in Figure 13a, similar to the cleaning of hard attachments, the variation trends of the three spectral lines C II-472.741 nm, O II-658.9006 nm, and Al II-587.198 nm (hereafter referred to as C II, O II, and Al II) are the most significant and are used as key spectral lines for analysis. In contrast, Figure 13b shows these lines as non-key spectral lines.

As shown in Figure 13a, the three characteristic spectral lines increased with energy density. Below 7 J/cm^2^, the Al II intensity remained low. At the same time, C II and O II exceeded 500, indicating that only a small portion of the EPS layers had been ablated, leaving the substrate untouched. As more EPS layers were removed, plasma emission of C II and O II intensified, and Al II strengthened as the substrate became partially exposed. At approximately 10 J/cm^2^, all three spectral lines stabilized.

**Figure 14:**
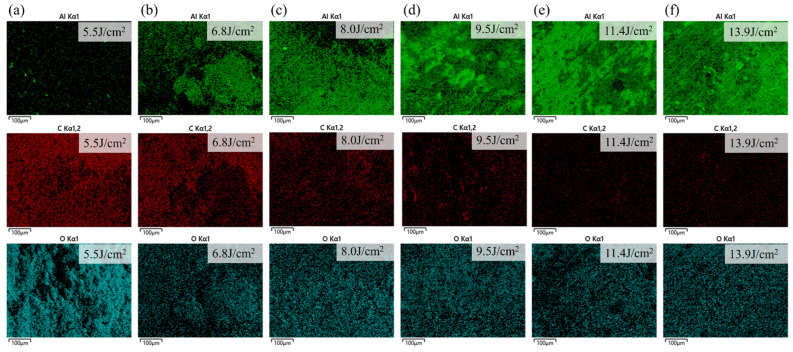
EDS scanning results of Al, C, and O elements on the surface of EPS layers after cleaning with different energy densities. (**a**) 5.5 J/cm^2^; (**b**) 6.8 J/cm^2^; (**c**) 8.0 J/cm^2^; (**d**) 9.5 J/cm^2^; (**e**) 11.4 J/cm^2^; (**f**) 13.9 J/cm^2^.

The EDS surface scans of Al, C, and O after cleaning are shown in Figure 14. Al content increased with energy density, while C content decreased. O content first declined, reaching its lowest point (28.72 At.%) at 6.8 J/cm^2^, and then stabilized before rising again due to substrate oxidation as energy density increased. At 8.0 J/cm^2^, the Al covered most of the surface, the C was significantly reduced, and the O content stabilized, indicating the effective removal of EPS layers; at this point, the atomic percentage of Al was 44.32%. As energy density continued to increase, the Al and C contents gradually stabilized, and their distribution became more uniform, suggesting that the laser had fully interacted with the substrate, leading to substrate oxidation.

### 3.4. Pearson Correlation of “Reference Spectra” to Assess Cleaning Effectiveness

As discussed in Section 3.3, the optimal laser energy densities for cleaning hard attachments and EPS layers were 11.4 J/cm^2^ and 8.0 J/cm^2^, respectively. At these levels, the biofilm layers were almost entirely removed, exposing the substrate surface and yielding a well-cleaned sample. The *CE* plasma spectra obtained under these optimal cleaning conditions were defined as “reference spectra”. The effectiveness of surface cleaning was then evaluated by calculating the Pearson correlation between the spectra from randomly cleaned surfaces and the “reference spectra”. The Pearson correlation quantified the linear relationship between a given spectrum and the “reference spectrum”, focusing on trends in spectral variations rather than absolute intensity, which made it a straightforward and efficient approach for analysis. The Pearson correlation between two spectral datasets was calculated using Equation (2). In this section, we calculated the Pearson correlation between the specific peaks of each microbial layer and the reference spectrum to evaluate the cleaning effectiveness, specifically focusing on Ca I, Ca II, and Al II in the hard attachments, as well as C II, O II, and Al II in the EPS layer. The reference spectrum was obtained by conducting ten experiments under optimal process conditions and averaging the intensities of the selected spectral lines. Ultimately, the intensities of three spectral lines were chosen as the reference spectrum.

(2) $$R=\frac{\sum_{i=1}^{n}(x_{i}-\bar{x})(y_{i}-\bar{y})}{\sqrt{\sum_{i=1}^{n}(x_{i}-\bar{x}{)}^{2}\sqrt{\sum_{i=1}^{n}(y_{i}-\bar{y}{)}^{2}}}}$$

**Figure 15:**
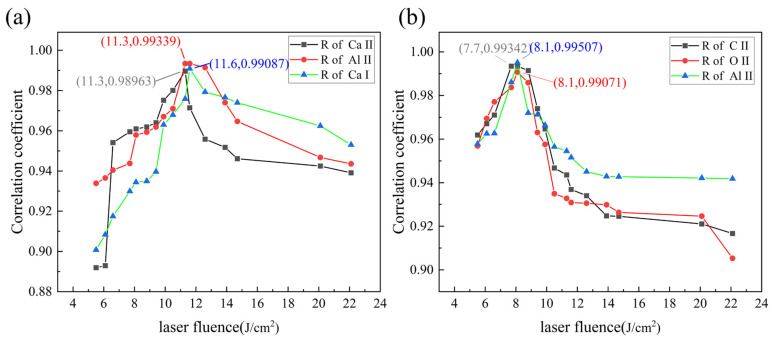
Correlation coefficients between random spectra and ‘reference spectra’ when cleaning the biofilm layers. (**a**) Hard attachments. (**b**) EPS layers.

Pearson’s correlation coefficient, which was closer to 1, indicated a closer match between the plasma spectrum and the spectrum under optimal cleaning conditions. Figure 15 shows the Pearson correlation coefficients for CE plasma spectra at different energy densities when cleaning hard attachments and EPS layers, compared to the reference spectrum. For hard attachment cleaning, the correlation coefficients of the three spectral lines increased, peaked, and then declined, with the highest correlation occurring at the inflection point. The highest correlation between the random spectral line and the reference spectrum determines the predicted value of the optimal energy density, which corresponds to the inflection point in the figure. The Al II and Ca II spectral lines reached this point at 11.3 J/cm^2^, indicating an optimal cleaning effect at this energy density. This value was taken as the predicted optimal cleaning parameter, with a relative error of approximately 0.9%. The Ca I spectral line reached its inflection point at 16 J/cm^2^, with a relative error of about 1.8%. For EPS layer cleaning, the correlation coefficients of the three spectral lines followed a similar trend. The O II and Al II spectral lines peaked at 8.1 J/cm^2^, with a relative error of about 1.2%, while the C II spectral line reached its highest correlation at 7.7 J/cm^2^, with a relative error of about 3.8%.

**Figure 16:**
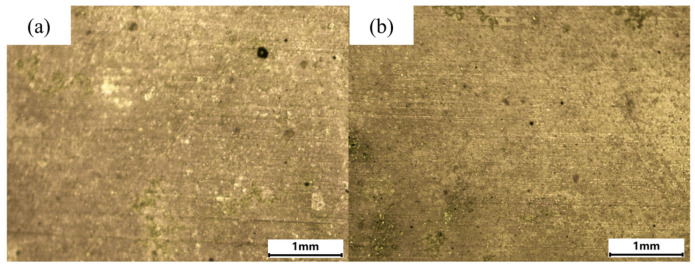
Macroscopic morphology of the surface after cleaning the microbial layer under the predicted parameters. (**a**) Hard attachments. (**b**) EPS layers.

The macroscopic morphology of the surface after cleaning at the predicted optimal parameters is shown in Figure 16. When cleaned at the highest correlation parameters, both the hard attachments and EPS layers were completely removed, exposing the metal substrate. When the energy density was lower than predicted, the correlation decreased, and the surface microbial layer could not be removed completely, and when the energy density was higher than predicted, the correlation similarly decreased, and the biofilm layers could be removed while harming the surface of the substrate and producing oxidation. In summary, the correlation between random spectra and “reference spectra” can be used to analyze and predict the surface removal of different biofilm layers.

## 4. Conclusions

In this paper, a nanosecond pulsed laser was used to clean different marine biofilm layers from the surface of aluminum alloy. A spectrometer was employed to monitor plasma during the cleaning process, and the plasma spectra of the two biofilm layers were extracted within the 300–800 nm wavelength range. The relationship between the spectral evolution patterns in the long-wavelength region and the cleaning effectiveness was analyzed. Furthermore, regression fitting analyses were performed on the intensities of plasma spectral lines in specific bands of *CE* for both microbial layers to characterize elemental changes and evaluate the cleaning effect on the material surface.

(1) The spectral characteristics of different microbial layers during laser cleaning were analyzed. The *CE* of the hard attachments was selected as Ca, and the *CE* of the EPS layers was selected as C and O by analyzing the EDS spectra. Under laser irradiation, the results of the spectral line calibration of hard attachments showed high-intensity Ca atomic lines and singly ionized lines, in addition to low-intensity C, O, Al, and other spectral lines. The spectral line calibration results of the EPS layers had a large number of high-intensity C and O atomic spectral lines and singly ionized lines, in addition to a small number of Al and Fe spectral lines, which indicated that different biofilm layers mainly radiated high-intensity *CE* spectral lines under laser action and the intensity of the spectral lines changed with different cleaning states.

(2) The effect of laser cleaning energy on the intensity of CE spectral lines was investigated, and the optimal cleaning process for the two biofilm layers was determined. Nonlinear curve fitting was performed to analyze the relationship between spectral line intensity at specific wavelengths and laser energy density. The EDS surface spectral scanning results after cleaning indicated that the optimal energy densities for removing hard attachments and EPS layers were 11.4 J/cm^2^ and 8.0 J/cm^2^, respectively. At these energy levels, the cleaning process effectively eliminated the surface biofilm layers, exposed the aluminum substrate, and achieved a satisfactory cleaning outcome.

(3) The plasma spectral lines of well-cleaned *CE* were defined as the “reference spectrum”. The Pearson correlation coefficient was calculated to evaluate the correlation between randomly acquired spectra and the “reference spectrum”, which indicated that the process corresponding to the random spectrum with the highest correlation deviated from the optimal cleaning parameters by approximately 0.9% to 3.8%. The finding demonstrated that the correlation between random spectra and the “reference spectrum” can effectively predict the optimal cleaning conditions for specific biofilm layers, providing a foundation for the further development of spectral-based intelligent feedback control in laser cleaning.

Automated intelligent laser cleaning is expected to be a key development trend in the future. Intelligent recognition of the real-time cleaning state and the incorporation of a feedback control system are crucial. Our study provides a preliminary foundation for the intelligent identification of marine biofouling removal quality on aluminum alloy surfaces, offering theoretical guidance and direction for future research. In addition, the implementation of a feedback control system is particularly important for realizing intelligent laser cleaning.

## Figures and Tables

**Table 1:** Chemical compositions of 6061 aluminum alloy (wt.%).

Element	Si	Mg	Cu	Fe	Mn	Al
Content	0.40–0.80	0.8–1.2	0.15–0.4	≤0.7	≤0.15	Balance

## Data Availability

The original contributions presented in this study are included in the article. Further inquiries can be directed to the corresponding author.
